# The grade of individual prostate cancer lesions predicted by magnetic resonance imaging and positron emission tomography

**DOI:** 10.1038/s43856-023-00394-7

**Published:** 2023-11-09

**Authors:** Erik Nilsson, Kristina Sandgren, Josefine Grefve, Joakim Jonsson, Jan Axelsson, Angsana Keeratijarut Lindberg, Karin Söderkvist, Camilla Thellenberg Karlsson, Anders Widmark, Lennart Blomqvist, Sara Strandberg, Katrine Riklund, Anders Bergh, Tufve Nyholm

**Affiliations:** 1 Department of Radiation Sciences, Radiation Physics, Umeå University, Umeå, Sweden; 2 Department of Radiation Sciences, Oncology, Umeå University, Umeå, Sweden; 3 Department of Molecular Medicine and Surgery, Karolinska Institutet, Solna, Sweden; 4 Department of Radiation Sciences, Diagnostic Radiology, Umeå University, Umeå, Sweden; 5 Department of Medical Biosciences, Pathology, Umeå University, Umeå, Sweden

**Keywords:** Cancer imaging, Magnetic resonance imaging, Radionuclide imaging, Prostate cancer

## Abstract

**Background:**

Multiparametric magnetic resonance imaging (mpMRI) and positron emission tomography (PET) are widely used for the management of prostate cancer (PCa). However, how these modalities complement each other in PCa risk stratification is still largely unknown. We aim to provide insights into the potential of mpMRI and PET for PCa risk stratification.

**Methods:**

We analyzed data from 55 consecutive patients with elevated prostate-specific antigen and biopsy-proven PCa enrolled in a prospective study between December 2016 and December 2019. [^68^Ga]PSMA-11 PET (PSMA-PET), [^11^C]Acetate PET (Acetate-PET) and mpMRI were co-registered with whole-mount histopathology. Lower- and higher-grade lesions were defined by International Society of Urological Pathology (ISUP) grade groups (IGG). We used PET and mpMRI data to differentiate between grades in two cases: IGG 3 vs. IGG 2 (case 1) and IGG ≥ 3 vs. IGG ≤ 2 (case 2). The performance was evaluated by receiver operating characteristic (ROC) analysis.

**Results:**

We find that the maximum standardized uptake value (SUV_max_) for PSMA-PET achieves the highest area under the ROC curve (AUC), with AUCs of 0.72 (case 1) and 0.79 (case 2). Combining the volume transfer constant, apparent diffusion coefficient and T2-weighted images (each normalized to non-malignant prostatic tissue) results in AUCs of 0.70 (case 1) and 0.70 (case 2). Adding PSMA-SUV_max_ increases the AUCs by 0.09 (*p* < 0.01) and 0.12 (*p* < 0.01), respectively.

**Conclusions:**

By co-registering whole-mount histopathology and in-vivo imaging we show that mpMRI and PET can distinguish between lower- and higher-grade prostate cancer, using partially discriminative cut-off values.

## Plain language summary

Magnetic Resonance Imaging (MRI) and Positron Emission Tomography (PET) are two medical imaging methods commonly used to image prostate cancers. However, the relationship between images obtained with these methods and prostate cancer aggressiveness is not well understood. Here, we investigate whether MRI and PET can differentiate between lower- and higher-grade prostate tumors, where grade is an indicator of how aggressive the disease is likely to be. We find that the characteristics of prostate cancer tumors as seen on MRI and PET scans can help to predict tumor grade. This means that these imaging methods may be helpful when clinicians are predicting patient prognosis and deciding on appropriate treatments. However, further validation is necessary before these approaches are widely implemented for this purpose.

## Abstract (web summary)

Nilsson et al. distinguish between lower- and higher-grade prostate cancers using imaging and whole-slide histopathology in a co-registered dataset. They find that clinically significant distinctions of prostate cancer grades are reflected in partially discriminative cut-off values derived from multiparametric MRI and positron emission tomography.

## Introduction

Multiparametric magnetic resonance imaging (mpMRI) is extensively used in the clinical management of prostate cancer (PCa) and is often reported according to the Prostate Imaging Reporting and Data System (PI-RADS) version 2.1. This scoring system consists of T2-weighted imaging (T2w), diffusion-weighted imaging (DWI) including apparent diffusion coefficient (ADC) maps, and dynamic contrast enhanced (DCE) sequences. Higher PI-RADS scores indicate a greater risk of clinically significant cancer and are associated with focal regions showing hypointense signal on T2w, hypointense signal on ADC, hyperintense signal on DWI and early enhancement on DCE^1^.

The signal in DWI relates to how the random motion of water is restricted and can be quantified by ADC maps, where hypointense regions have been shown to correlate with increased cell density^2^ and prognostic markers such as Gleason scores^3,4^. DCE can be evaluated quantitatively by modelling the transport of contrast agent from blood vessels to surrounding tissues via a set of pharmacokinetic parameters. One of these parameters is the volume transfer constant (K^trans^) which reflects the transfer rate of the contrast agent and has been associated with tumor angiogenesis^5,6^.

Positron emission tomography (PET) with tracers such as [^68^Ga]PSMA-11 (PSMA-PET) and [^11^C]Acetate (Acetate-PET) provides molecular information of the pathological changes caused by PCa. [^68^Ga]PSMA-11 targets prostate-specific membrane antigen which is overexpressed in PCa cells^7^ and higher maximum standardized uptake values (SUV_max_) have been associated with worse outcomes^8,9^. Accumulation of [^11^C]Acetate relates to the increased fatty acid synthesis driven by the overexpression of fatty acid synthase in PCa cells, which has been correlated with more aggressive forms of PCa^10^ and worse prognosis following biochemical relapse after prostatectomy^11^.

Given the extensive structural and functional diagnostic information provided by the mpMRI, and the molecular functional information from the PET, the combination of these modalities has the potential to further improve diagnostic imaging in PCa^12^. The hybrid imaging solution allows for integrated PET and MRI in the same scanner at the same timepoint. Simultaneous PET/MRI data acquisition with software-based co-registration and image post-processing optimize the conditions for integrated hybrid reading and reporting.

Some of the most commonly used prognostic markers for PCa are derived from histologic grading of resection and biopsy specimens^13^. The Gleason grading system^14^ has been revised over the years, and in addition to Gleason scores, the International Society of Urological Pathology (ISUP) now also recommends reporting ISUP grade groups (IGG)^15^. IGG is a five-grade group system (1–5) and is in part intended to address the observed variability in the clinical outcome for patients with Gleason score 7 = 3 + 4 (IGG 2) and Gleason score 7 = 4 + 3 (IGG 3)^16–19^.

While mpMRI and PET have been shown to reflect the severity of PCa^8–12,20,21^, it is less well known how multiple image modalities complement each other in PCa risk stratification.

The aim of this study was to investigate the potential to differentiate between ISUP Grade Groups using mpMRI, PSMA-PET and Acetate-PET, with whole-mount registered histopathology as reference standard. The evaluation was limited to the discrimination between higher- and lower-grade lesions (IGG 3 vs. IGG 2 and IGG ≥ 3 vs. IGG ≤ 2).

In this work, we show that the clinically significant differentiation between ISUP grade group 2 and 3 is reflected in partially discriminative cut-off values derived from PSMA-PET/mpMRI and Acetate-PET. The results indicate that PSMA-SUV_max_ is the most informative quantitative image measure, and that it provides independent information to mpMRI-based modalities. Out of the mpMRI-based modalities, image summary measures derived from K^trans^-images are in our data the most informative, with AUCs closer to PSMA-SUV_max_ than other modalities. Meanwhile, ADC- maps and T2w images contribute less to the distinction between ISUP grade groups.

## Patients and methods

### Study population

Patient characteristics are detailed in Table 1. This observational study was approved by the Regional Ethics Board and the Radiation Protection Committee at Umeå University Hospital (EudraCT number: 2015-005046-55). Fifty-five consecutive patients (median age: 63 years; range: 45–76 years) were enrolled between December 2016 and December 2019. All patients had elevated prostatic-specific antigen (PSA) (median PSA: 6.3 ng/ml; range: 2.9–13.3 ng/ml), biopsy-verified intermediate and high-risk PCa (IGG ≥ 2, at least 2 months prior to surgery), and were planned for laparoscopic radical prostatectomy. The patients were examined with PSMA-PET/mpMRI and Acetate-PET/CT prior to surgery, after providing written informed consent (Regional Ethics Board approval: Dnr 2016-220-31 M). The median time between imaging and surgery was 26 days (range: 2–138 days). PET/mpMRI and PET/CT was completed in a single day for most patients (49/55), and the maximum time between imaging procedures was 1 month for the remaining six patients.

**Table 1:** Patient characteristics.

Variable	Median (range)
Patients (n)	55
PSA (ng/ml)	6.3 (2.9–13.3)
PSA density (ng/ml^2^)	0.16 (0.06–0.46)
Age (years)	63 (45–76)
Time between PET/mpMRI and PET/CT (days)	1 (1–31)
Time between imaging and surgery (days)	26 (2–138)
Post RP ISUP	
2	29
3	17
4	5
5	4
pT status	
T2	24
T3	31
pN status	
Not removed	44
Lymph nodes removed without metastasis	9
Lymph nodes removed with metastasis	2

*PSA* prostate-specific antigen, *PET* positron emission tomography, *mpMRI* multiparametric magnetic resonance imaging, *CT* computed tomography, *RP* radical prostatectomy, *ISUP* International Society of Urological Pathology.

### PSMA-PET/mpMRI

PSMA-PET/mpMRI was acquired with a 3.0 T PET/MRI system (Signa; GE Healthcare, Waukesha, WI, USA). The mpMRI included T2w, DWI and DCE MRI sequences (Table 2). Fast spin-echo T2w images were obtained in three planes (axial, coronal, sagittal). Echo-planar DWI was performed with b-values of b_0_ = 0 s mm^−2^, b_200_ = 200 s mm^−2^ and b_1000_ = 1000 s mm^−2^. ADC maps were calculated by using the monoexponential decay model with two measurements (b_200_ and b_1000_). All image processing was done using MICE Toolkit (NONPI Medical, Umeå, Sweden) unless otherwise stated. See Supplementary Methods for further details.

**Table 2:** PSMA-PET/mpMRI and Acetate-PET parameters.

	FA (°)	MS (px)	Avgs.(No.)	TE (ms)	TR (ms)	PS (mm)	ST (mm)
DCE	20	256 × 256	1	1.82–1.90	4.03–4.49	1.00/1.00	5.0
DWI	90	256 × 256	4	73.0–73.5	3500–4500	0.94/0.94	5.0
T2w (Ax)	125	512 × 512	1	95.7–104.3	3730–10,941	0.41/0.41	2.5
T2w (Sag)	111	512 × 512	3	125.2–134.9	4922–7482	0.47/0.47	3.0
T2w (Cor)	111	512 × 512	3	125.2–134.9	6133	0.47/0.47	3.0
T2w ex-vivo	111	512 × 512	15	117	2500	0.20/0.20	5.0
VFA	2, 15	256 × 256	2	1.776	4.892–4.908	1.02/1.02	5.0
PSMA-PET	–	256 × 256	–	–	–	2.34/2.34	2.8
Acetate-PET	–	256 × 256	–	–	–	2.73/2.73	3.3

*DCE* dynamic contrast enhanced imaging, *DWI* diffusion-weighted imaging, *Ax* transaxial, *Sag* sagittal, *Cor* coronal, *T2w* T2-weighted, *VFA* variable flip angle, *PSMA* prostate-specific membrane antigen, *PET* positron emission tomography. *FA* flip angle, *MS* matrix size, *Avgs.* no. averages, *TE* echo time, *TR* repetition time, *PS* in-plane pixel size, *ST* slice thickness.

DCE images were obtained as 50 frames over 8 min by a Fast Spoiled Gradient Recalled Echo (FSPGR) T1-weighted (T1w) sequence with 0.2 ml/kg intravenously injected gadolinium (GD)-based contrast agent (Dotarem, 279.3 mg/ml, Guerbet, Villepinte, France). The DCE frames were motion corrected and used in a three-parameter Kety model to calculate K^trans^^22^. The Kety model was implemented with patient-specific T1-maps and arterial input functions (AIFs). The AIFs were determined from manually delineated ROIs in the deep and/or superficial femoral arteries for each patient, and T1-maps were estimated by the variable-flip angle (VFA) method (2° and 15°)^23^.

[^68^Ga]PSMA-11 was synthesized in-house^24^, and 2.0 MBq/kg was injected intravenously (median injected activity: 163 MBq; range: 121–201 MBq). PSMA-PET data was acquired from one bed position covering the whole pelvic region and reconstructed using a 3D ordered subset expectation maximization algorithm with resolution recovery (SharpIR; GE Healthcare, Waukesha, WI, USA). The acquisition was initiated 60 min post injection, lasted for 40 min, and was completed during the MRI sequences.

### Acetate-PET/CT

Acetate-PET was acquired on a PET/CT system (Discovery 690; GE Healthcare, Waukesha, WI, USA), starting with a low-dose CT for attenuation correction and a diagnostic-quality CT. PET-data acquisition was started 10 min post injection of 5 MBq/kg [^11^C]Acetate (median injected activity: 426 MBq; range: 286–544 MBq). PET was acquired from proximal femur to the head using time-of-flight, 2 min/bed position and 11 slices overlap. The images were reconstructed to a 70 cm field-of-view, using the SharpIR reconstruction algorithm with three iterations, 24 subsets and a 3.0 mm post filter.

### Histopathological preparation and evaluation

**Fig. 1:**
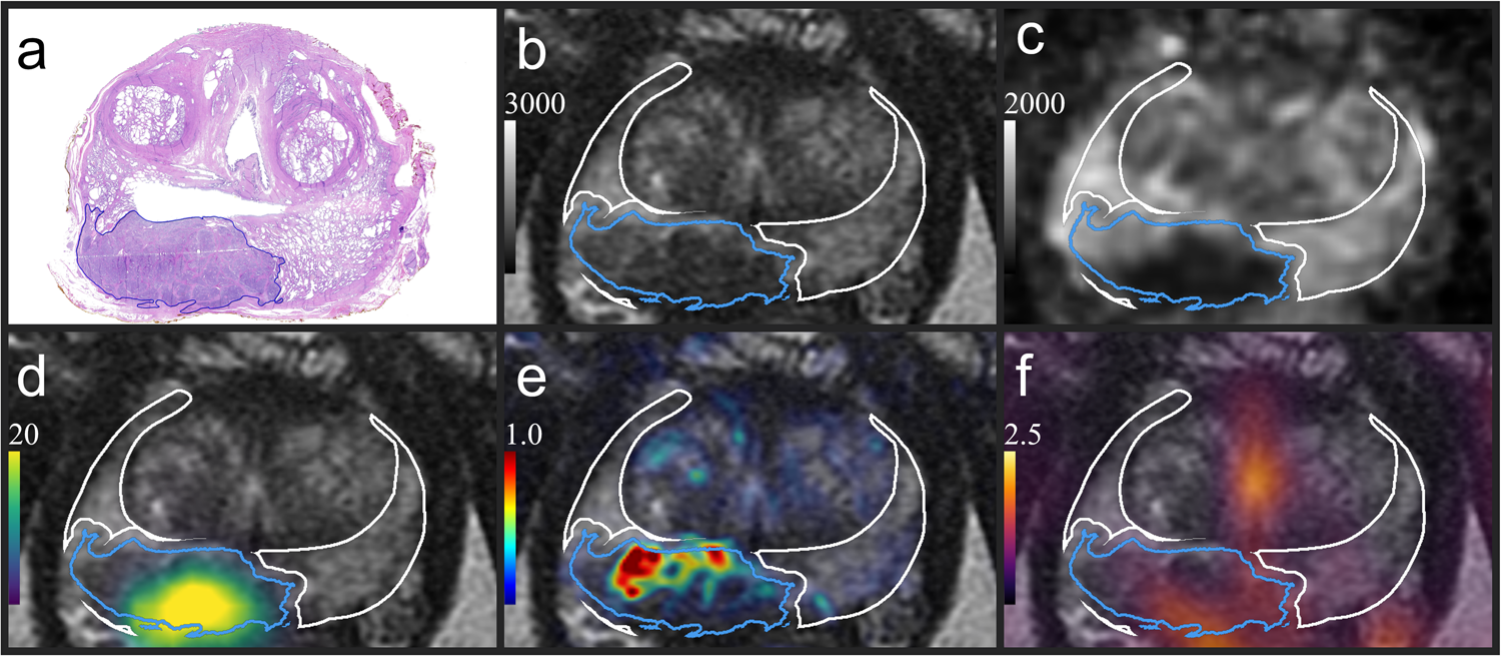
Co-registered image data. **a** Histological section showing the contour of an IGG 4 lesion (blue). **b** T2w with the registered lesion and the non-malignant PZ (white), where voxels in the PZ closer than 1 mm from lesions had been removed. The T2w image served as a common frame of reference for the histopathology, ADC [µm^2^ s^−1^] (**c**), SUV PSMA-PET [g/ml] (**d**), K^trans^ [min^−1^] (**e**) and SUV Acetate-PET [g/ml] (**f**). See Supplementary Fig. 1 for a more nuanced example. IGG International Society of Urological Pathology grade group, T2w T2-weighted, PZ peripheral zone, ADC apparent diffusion coefficient, PSMA prostate-specific membrane antigen, SUV standardized uptake value, PET positron emission tomography, K^trans^ volume transfer constant.

Prostates were contoured on the T2w image, and a 3D printed mold was tailored for each individual prostate based on these delineations^25^. Following surgery, the prostate was placed inside its mold and scanned to yield ex-vivo T2w images of the prostate. The prostates were then prepared for histopathological evaluation. First formalin-fixed, then sectioned in the mold into 5 mm blocks. These blocks were then dehydrated and paraffin-embedded. A 5 µm thick microtome section was taken from each block, such that the sections coincided with the ex-vivo slices. The microtome sections were first evaluated clinically by a board-certified pathologist (A.B., with >30 years of experience) and then digitally scanned (NanoZoomer-XR C12000; Hamamatsu Photonics, Hamamatsu, Shizuoka, Japan). Based on the initial evaluations, detailed digital annotations on the scanned microtome sections were provided by A.K.L. (PhD) under supervision and final approval of A.B., resulting in regions of interest (ROIs) with IGGs (Fig. 1a). For brevity, we refer to these ROIs as lesions.

### Co-registration

The mpMRI, PSMA-PET, Acetate-PET and histopathology were aligned with the in-vivo T2w image. The prostatic volume of the DWI(b_0_) was reshaped to match the prostatic volume of the T2w using a non-rigid registration method. The resulting transformations were applied to the remaining two DWIs, and the ADC was calculated from the registered DWI(b_200_) and DWI(b_1000_). K^trans^ was registered by the same procedure as was explained for the DWI(b_0_). The CT was registered to the T2w using a rigid registration process, and the resulting transform was applied to the Acetate-PET data. PSMA-PET data was assumed to be aligned with the T2w image. The scanned and annotated microtome sections were non-rigidly registered to the in-vivo T2w, using affine registrations to the ex-vivo images as an intermediary step. See Supplementary Methods for additional registration- and imaging details.

### Image analysis

The current analysis of PSMA-PET/mpMRI image data was performed in two steps. First, the anatomical contour of the prostate gland was outlined by two medical physicists (J.J. & K.S.) and verified by a double-licensed radiologist and nuclear medicine physician (S.S., with >10 years of experience) for definition of region of interest. The image data was derived from previously unreported study examinations. In a subsequent radiological reporting (not covered in this study), MRI image quality was assessed in grades 1–4: 1=poor; 2=fair (diagnostic); 3=good; 4=excellent. For the present study, no images were excluded due to poor image quality.

In a second step, the prostate zones were delineated on the in-vivo T2w using RayStation version 8.99.30 (RaySearch Laboratories, Stockholm, Sweden). From the peripheral zone (PZ) we defined a non-malignant PZ by excluding voxels within the PZ closer than 1 mm from the registered lesions, see Fig. 1b. The buffer zones surrounding the lesions were added to account for registration uncertainties which reduces the risk of inadvertently including malignancies in the non-malignant PZ.

Tumors appearing in multiple slices were treated as independent lesions. We extracted five values for each lesion: The PET-data for each lesion was quantified by SUV_max_ for both Acetate-PET and PSMA-PET. The mpMRI was quantified by taking the median ADC, median intensity from the T2w image and maximum K^trans^, and dividing by the corresponding mean intensities in the non-malignant PZ taken voxel-wise per patient. We refer to these five values as modalities, see Table 3.

**Table 3:** Definition of modalities used in the ROC analysis.

Modality name	Description
PSMA	SUV_max_ [g/ml] per lesion for [^68^Ga]PSMA-11
ACE	SUV_max_ [g/ml] per lesion for [^11^C]Acetate
$\widetilde{{{\mbox{K}}}}$^trans^	Maximum K^trans^ [min^−1^] per lesion, divided by the mean K^trans^ in the non-malignant PZ of the patient
$\widetilde{{{\mbox{ADC}}}}$	Median ADC [µm^2^ s^−1^] per lesion, divided by the mean ADC in the non-malignant PZ of the patient
$\widetilde{{{\mbox{T}}}2}$	Median image intensity per lesion in the T2w image, divided by the mean image intensity in the non-malignant PZ of the patient

*ROC* receiver operating characteristic, *PSMA* prostate-specific membrane antigen, *SUV*_*max*_ maximum standardized uptake value, *K*^*trans*^ volume transfer constant, *ADC* apparent diffusion coefficient, *T2w* T2-weighted, *PZ* peripheral zone.

Tilde signifies a ratio, making these quantities unitless.

### Statistics and reproducibility

The modalities were used alone, or in combination, to predict ISUP Grades for lesions with a histopathologically defined in-plane area greater than or equal to 20 mm^2^ (*n* = 194 independent lesions for IGG ≥ 3 vs. IGG ≤ 2; *n* = 123 for IGG 3 vs. IGG 2). Receiver operating characteristic (ROC) analysis were used for each modality to find the area under the ROC curve (AUC) and the Youden thresholds, defined as the cut-off values yielding highest Youden index (sensitivity + specificity – 1). For combination of modalities, we fitted logistic regression models with the modalities as individual variables. AUCs were compared using two-tailed *p* values following a fast implementation of DeLong’s algorithm^26^. We did not correct for multiple testing, which increases the risk of false positive findings and mitigates the risk of being overly conservative. It is important to bear this in mind when interpreting the results. Data to correct for multiple testing can be obtained from Supplementary Figs. 2–3.

We fitted univariate logistic regression models to adjust each modality for lesion size. All *p* values < 0.05 were considered statistically significant. See Supplementary Methods for additional details of the classification algorithm.

## Results

Lesion characteristics are summarized in Table 4. In total, 600 lesions were identified. The results are based on the 194 lesions having an in-plane area ≥20 mm^2^.

**Table 4:** Lesions characteristics.

ISUP grade group	*n* (%)	*n* ≥ 20 mm² (%)	Estimated location [%], all (area ≥ 20 mm²)			
			PZ	TZ	CZ	AFS
1	304 (51)	40 (21)	55 (40)	36 (45)	6 (10)	3 (5)
2	131 (22)	74 (38)	66 (55)	24 (28)	9 (14)	2 (3)
3	72 (12)	49 (25)	82 (82)	12 (14)	6 (4)	0 (0)
4	63 (10)	19 (10)	52 (58)	19 (21)	25 (21)	3 (0)
5	30 (5)	12 (6)	53 (75)	33 (17)	13 (8)	0 (0)

Number of lesions (percentages) and estimated location per IGG and corresponding figures for lesions having a histopathologically defined in-plane area ≥ 20 mm². The location corresponds to the percentage of cases where a specific zone accommodated the largest portion of the lesion.

*ISUP* International Society of Urological Pathology, *PZ* peripheral zone, *TZ* transition zone, *CZ* central zone, *AFS* anterior fibromuscular stroma.

The ROC analysis is summarized in Table 5 and Table 6, where a correctly classified higher-grade lesion was considered a true positive. The ROC curves are shown in Supplementary Fig. 4. PSMA achieved the highest AUC of all modalities, with an AUC of 0.72 for IGG 3 vs. IGG 2 and 0.79 for IGG ≥ 3 vs. IGG ≤ 2. In the former case, however, AUC-PSMA was only significantly higher than AUC-$\widetilde{{{\mbox{T}}}2}$ and AUC-ACE, while in the latter case AUC-PSMA was significantly higher than all other modalities (Table 6). Combining the biparametric MRI-based modalities ($\widetilde{{{\mbox{ADC}}}}$ and $\widetilde{{{\mbox{T}}}2}$) as individual variables in logistic regression models resulted in an AUC of 0.66 for IGG 3 vs. IGG 2 and 0.61 for IGG ≥ 3 vs. IGG ≤ 2. The corresponding figures for the mpMRI-based modalities ($\widetilde{{{\mbox{K}}}}$^trans^, $\widetilde{{{\mbox{ADC}}}}$ and $\widetilde{{{\mbox{T}}}2}$) were 0.70 and 0.70, with *p* < 0.01 for the latter increase. PSMA combined with the mpMRI-based modalities as variables in logistic regression models further increased the AUCs to 0.79 (*p* < 0.01) and 0.82 (*p* < 0.01), respectively. In univariate logistic regression, all modalities except for $\widetilde{{{\mbox{T}}}2}$ were significant predictors of IGG in the case of IGG 3 vs. IGG 2, and all but $\widetilde{{{\mbox{T}}}2}$ and $\widetilde{{{\mbox{ADC}}}}$ for the case of IGG ≥ 3 vs. IGG ≤ 2. Contrarily, size was not a significant predictor of IGG in either case. Adjusting each modality for size revealed that only $\widetilde{{{\mbox{K}}}}$^trans^ and PSMA were independent predictors of IGG (*p* < 0.01).

**Table 5:** Results of the ROC analysis.

Modalities	AUC	95% CI	Youden thresholds	Sensitivity (%)	Specificity (%)
$\widetilde{{{\mbox{T}}}2}$	0.48 (0.49)	38–58 (41–57)	0.90 (0.90)	78 (72)	35 (35)
$\widetilde{{{\mbox{ADC}}}}$	0.60 (0.58)	50–71 (49–66)	0.85 (0.85)	67 (62)	54 (55)
ACE	0.61 (0.59)	51–71 (51–67)	3.4 (4.3)	67 (44)	57 (75)
$\widetilde{{{\mbox{K}}}}$^trans^	0.68 (0.70)	58–78 (63–78)	4.7 (4.7)	53 (51)	82 (84)
PSMA	0.72 (0.79)	63–82 (72–86)	6.5 (6.5)	63 (68)	82 (87)
MRI_2_	0.66 (0.61)	57–76 (53–69)	–	–	–
MRI_3_	0.70 (0.70)	61–80 (63–78)	–	–	–
PSMA + MRI_3_	0.79 (0.82)	71–87 (76–88)	–	–	–

Figures in parentheses refer to IGG ≥ 3 vs. IGG ≤ 2, as opposed to IGG 3 vs. IGG 2.

PSMA, ACE, $\widetilde{{{\mbox{K}}}}$^trans^, $\widetilde{{{\mbox{ADC}}}}$ and $\widetilde{{{\mbox{T}}}2}$ as explained in Table 3; MRI_2_ = fitting a logistic regression model combining $\widetilde{{{\mbox{ADC}}}}$ and $\widetilde{{{\mbox{T}}}2}$ as individual variables; MRI_3_ = combining $\widetilde{{{\mbox{K}}}}$^trans^, $\widetilde{{{\mbox{ADC}}}}$ and $\widetilde{{{\mbox{T}}}2}$ as individual variables; PSMA + MRI_3_ = combining PSMA and the components of MRI_3_; AUC = area under the receiver operating characteristic (ROC) curve; CI = confidence interval for the AUCs; *n* = 194 independent lesions for IGG ≥ 3 vs. IGG ≤ 2 and *n* = 123 for IGG 3 vs. IGG 2; IGG International Society of Urological Pathology grade group; Youden thresholds = cut-off values resulting in the largest Youden index (sensitivity + specificity − 1). The reported sensitivities and specificities correspond to the Youden thresholds. See Supplementary Table 1 for the complete list of combinations.

**Table 6:** Comparison of ROC performances.

Modalities	ΔAUC	
	IGG 3 vs. IGG 2	IGG ≥ 3 vs. IGG ≤ 2
PSMA + MRI_3_ vs. MRI_3_	0.09**	0.12**
MRI_3_ vs. MRI_2_	0.04	0.09**
PSMA vs. MRI_2_	0.06	0.18**
PSMA vs. $\widetilde{{{\mbox{K}}}}$^trans^	0.04	0.09*
PSMA vs. ACE	0.11*	0.20**
PSMA vs. $\widetilde{{{\mbox{ADC}}}}$	0.12	0.21**
PSMA vs. $\widetilde{{{\mbox{T}}}2}$	0.24**	0.30**
$\widetilde{{{\mbox{K}}}}$^trans^ vs. ACE	0.07	0.11*
$\widetilde{{{\mbox{K}}}}$^trans^ vs. $\widetilde{{{\mbox{ADC}}}}$	0.07	0.12*
$\widetilde{{{\mbox{K}}}}$^trans^ vs. $\widetilde{{{\mbox{T}}}2}$	0.20**	0.21**
ACE vs. $\widetilde{{{\mbox{ADC}}}}$	<0.01	0.01
ACE vs. $\widetilde{{{\mbox{T}}}2}$	0.13	0.11
$\widetilde{{{\mbox{ADC}}}}$ vs. $\widetilde{{{\mbox{T}}}2}$	0.13**	0.09

ΔAUC = differences in area under the receiver operating characteristic (ROC) curve; The asterisk (*) signifies *p* < 0.05 and two asterisks (**) *p* < 0.01, using two-sided *p* values for the ΔAUC; *n* = 194 independent lesions for IGG ≥ 3 vs. IGG ≤ 2 and *n* = 123 for IGG 3 vs. IGG 2. IGG = International Society of Urological Pathology grade group; PSMA, ACE, $\widetilde{{{\mbox{K}}}}$^trans^, $\widetilde{{{\mbox{ADC}}}}$ and $\widetilde{{{\mbox{T}}}2}$ as explained in Table 3; MRI_2_ = fitting a logistic regression model combining $\widetilde{{{\mbox{ADC}}}}$ and $\widetilde{{{\mbox{T}}}2}$ as individual variables; MRI_3_ = combining $\widetilde{{{\mbox{K}}}}$^trans^, $\widetilde{{{\mbox{ADC}}}}$ and $\widetilde{{{\mbox{T}}}2}$ as individual variables; PSMA + MRI_3_ = combining PSMA and the components of MRI_3_. See Supplementary Figs. 2–3 for the complete set of comparisons.

## Discussion

This study demonstrated that image intensity thresholds obtained from PSMA-PET, Acetate-PET and mpMRI (Table 3) have the potential to differentiate between lower-grade lesions (IGG 2 or IGG ≤ 2) and higher-grade lesions (IGG 3 or IGG ≥ 3). For instance, if a SUV_max_ greater than 6.5 for PSMA-PET had been used as a threshold for higher-grade lesions, about two thirds of the higher-grade lesions would be correctly classified, whereas less than one in five lower-grade lesions would be erroneously classified.

We were unable to demonstrate that $\widetilde{{{\mbox{T}}}2}$ could discriminate between ISUP grades. In fact, since the estimated AUCs were below 0.5, higher $\widetilde{{{\mbox{T}}}2}$ was more indicative of higher grades in our data. This is counter-intuitive to the expectation that higher PI-RADS scores are associated with hypointense regions on T2w images^1^. Since logistic regression does not take such a priori knowledge into account, the AUC for models including $\widetilde{{{\mbox{T}}}2}$ are presumably slightly overestimated.

We found a weak association between ADC and ISUP grades. The most recent systematic review indicate that ADC can have moderate accuracy in separating IGG ≤ 1 from IGG ≥ 2^4^. However, the reported correlations between Gleason scores and ADC vary, and the same review found the correlation to be only moderate for lesions in the peripheral zone, and weak for lesions in the transition zone. We suspect that some of the variation in the reported correlations can be explained by how lesions are localized on the ADC-maps. If instead of using registered histopathology as ground truth, the boundaries of lesions are shaped after hypointense regions on the ADC-map itself, the correlations are valid under the condition that the region already is hypointense.

The role of DCE as part of mpMRI is debated. The risk of missing clinically significant PCa is reportedly low even without DCE^27^. Eliminating this sequence would also reduce scan time, cost and risk for adverse events^1^. On the other hand, our results indicate that DCE could provide useful information for IGG predictions. Furthermore, we found that $\widetilde{{{\mbox{K}}}}$^trans^ was independently associated with IGG after adjusting for size in univariate logistic regression (*p* < 0.01). A possible explanation for this observation is that higher Gleason scores have been associated with the formation of new vascular structures^28^, and newly built blood vessels leak more blood into the surrounding tissue^29,30^. Considering the results of studies suggesting that DCE may improve the sensitivity for detecting PCa, the PI-RADS committee endorse further research before eliminating DCE^1,31^.

It should be noted that this study started prior to the publication of PI-RADS v2.1. The slice thicknesses for the DCE and DWI deviates from the recommended 3 mm and were instead chosen to optimize matching with the pathologic sections. Hence, the performances of $\widetilde{{{\mbox{ADC}}}}$ and $\widetilde{{{\mbox{K}}}}$^trans^ may be underestimated, especially for lesion diameters <5 mm. This is mitigated by the fact that the main results of this paper focus on lesions with radii ≥2.5 mm.

Recent studies have shown that PSMA-PET/CT and PSMA-PET/mpMRI can outperform mpMRI in the detection of primary- and metastatic PCa^12,32–34^, and to have the potential to impact the clinical management of patients^35^. This is likely attributed to the overexpression of binding sites in PCa cells compared to healthy tissue^36^. In the current study we showed that PSMA was one of the most informative modalities in discriminating between grade groups. We also found PSMA to be independently associated with IGG after adjusting for size (p < 0.0001). To that end, we add to the evidence that PSMA-PET could impact the treatment-decision making.

It should be mentioned that there are several PSMA-targeted radiotracers in use, labelled with [^68^Ga] or [^18^F]. We used [^68^Ga]PSMA-11 as was current clinical practice at the time of the study. A drawback with [^68^Ga]PSMA-11 is the urinary excretion, where high intensity uptake in the urinary bladder potentially may obscure adjacent pathological uptakes, for instance in central parts of the periurethral zone of the prostate or in perivesical lymph node metastases. The presently used radiotracer at our institution, [^18^F]PSMA-1007, has almost no urinary excretion but hepatobiliary excretion instead, causing higher uptakes in the hepatic region, and in addition to that, more unspecific bone uptakes as well. However, a recent meta-analysis by Evangelista et al.^37^ has concluded that all accessible PSMA radiotracers show excellent performance in staging of primary and recurrent prostate cancer, and that the availability should guide the choice of tracer. Moreover, there are ongoing studies investigating the diagnostic properties of PSMA-based ligands that could potentially combine the diagnostic capacity of PET with therapeutic radionuclides^38–40^.

This project has several limitations. First, our evaluation of the potential to correlate image data and histopathological grades can only be as reliable as the grading itself, which suffers from interobserver variability and reproducibility^15,41,42^. Furthermore, since the prostatic zones have distinct image characteristics, the correlations between image data and histology will be zone dependent^43^. Second, the dataset only included 55 patients from a single center, although the dataset is the largest of its kind to the best of our knowledge. Due to the limited size of the dataset, we did not search for image measures giving optimal results, and instead opted for measures that we found to be consistent with the literature^8,44–46^. However, quantifying ADC-maps and T2w images using measures based on minimum or near-minimum intensities would have been more consistent with the other measures. For this reason, we include results for the minimum and near-minimum (5th percentile) measures in Supplementary Table 2 and Supplementary Fig. 6, where they were shown to yield negligible differences. Third, we excluded lesions having an in-plane area less than 20 mm², which is the area of a circle having a radius r ≈ 2.5 mm, corresponding to roughly twice the uncertainty of the registration method^25^. We acknowledge that this decision introduces bias against small lesions. However, 20 mm² is still small when compared to the size that PI-RADS defines as clinically significant cancer (0.5 cm³)^1^, and the radius (2.5 mm) is comparable to the core length of 6 mm used in the PROMIS study to define clinically significant cancer in terms of size^47^. Moreover, we found that the AUCs remained stable despite variations of lesion size cut-offs (Supplementary Figs. 5–6). Fourth, we limited this study to focus on IGG 2 and IGG 3 lesions. This decision was in part motivated by the abundance of these lesions in our dataset. While it may reduce the applicability of the results to other grade groups, we can see that the distinction between IGG 2 and IGG 3 is particularly interesting, given the recent evidence in favor of considering active surveillance for patients having low amount of Gleason pattern 4 in absence of cribriform architecture or intraductal carcinoma^15,48–50^. Furthermore, the Swedish national guidelines on primary radiotherapy for PCa now recommends concomitant and adjuvant hormonal treatment for patients with unfavorable intermediate-risk PCa ( ≥ 50% positive biopsy cores, GS ≥ 4 + 3 and/or 2–3 intermediate-risk factors)^51,52^. This update is in line with the guidelines brought forth by the American Urology Association in collaboration with the American Society for Radiation Oncology^53^, and the growing body of literature in support of distinguishing between favorable- and unfavorable patient categories within the intermediate-risk group^18,19,54^. In addition, using IGG 3 or IGG ≥ 3 as the thresholds for higher-grade lesions in this work is similar to the PROMIS study, where they found no patients having clinically significant cancer in terms of grades (GS ≥ 4 + 3) when the mpMRI was negative (PI-RADS I/II)^47^.

One of the strengths of this study is its simplicity, which is expected to provide robustness to the classification algorithm. Furthermore, tissue-based normalization shows desirable properties as a harmonization technique, and may facilitate comparisons between our results and other studies^45^. Factors such as study design, scanner variability and inclusion criteria will affect the generalizability, underscoring the importance of further validation. Nonetheless, the methodology is not applicable in the clinical setting since we base our results on histopathologically defined lesions. Consequently, normalization by the non-malignant PZ is not clinically applicable.

The distinct zonal characteristics of the prostate suggest the potential for improved performance using zone-dependent normalization. We instead opted for an intensity normalization strategy that is less complex, since the handling of lesions extending into multiple zones is ambiguous and zone-specific optimization may be unnecessary for the purpose of this study. Another option could be normalization to non-prostatic tissue, for instance the obturator muscle^55,56^. In our dataset, positional correspondence outside the prostate is less reliable since the image registrations were focused on aligning prostatic regions (see Supplementary Methods). Similarly, we did not require an artificial system for combining lesions and their grades, as we relied on the histopathological evaluation, wherein each lesion was graded independently from other slices. This limits our results to slice-wise discrimination.

### Reporting summary

Further information on research design is available in the Nature Portfolio Reporting Summary linked to this article.

### Supplementary information

Supplementary Information

Reporting Summary

## Data Availability

The data that support the findings of this study are available from the corresponding author upon reasonable request.
