# Supplementary material for: The grade of individual prostate cancer lesions predicted by magnetic resonance imaging and positron emission tomography
Source: Commun Med (Lond). 2023 Nov 9;3:164. doi: 10.1038/s43856-023-00394-7 (PMC10636013; doi:10.1038/s43856-023-00394-7)
Supplement: Supplementary file 1 — Supplementary Information [file 43856_2023_394_MOESM1_ESM.pdf]

Supplementary Information

Supplementary Methods

Software and preprocessing

Prostate glands and prostatic zones were delineated on T2-weighted (T2w) images using RayStation v.8.99.30.16 (RaySearch Laboratories, Stockholm, Sweden) and exported as DICOM RT structure sets. All other image processing for this work was done in MICE Toolkit (Medical Interactive Creative Environment, NONPI Medical, Umeå, Sweden) [1]. This in-house developed software integrates the elastix [2] software package for image registration in a graphical programming user interface.

Registrations

Three main types of registrations have been used in this work. Translations and non-rigid registrations take advantage of delineations of the prostate, referred to as *regions of interest* (ROIs) in fixed (ROI_F) and moving (ROI_M) images.

Translation

The transformation $\mathcal{T}_T$ describes the translation between the geometrical mass centers of an ROI_M and an ROI_F. The origin-to-origin translation vector between the geometrical mass centers ($\Delta\mathbf{x}_R$) was then applied to the moving image.

Rigid registration

The transformation $\mathcal{T}_R$ explaining the mapping between the spatial domain of a moving and fixed image was found by a 3-D three-level multiresolution rigid registration method. We used a linear interpolator during optimization with *adaptive stochastic gradient descent* [3], nearest neighbour interpolation during resampling and random coordinate sampling. Here, 2048 samples were drawn from the fixed image for 300 iterations, using the *euler transform*. A *mean squared difference* (MSD) similarity metric was used when the inputs were binary masks, otherwise the *advanced mattes mutual information* was used [2].

Non-rigid registration

The non-rigid transformation $\mathcal{T}_N$ between a fixed and moving image was found using a 3-D non-rigid multimetric registration method. We used *local mutual information* (LMI) [4] and MSD as similarity measures for image data and *internal distance-preservation* (DP) [5], respectively. The measures were weighted 3:1 in favour of LMI and Algorithm 1 describes how DP-maps (DP_i) were determined for each ROI_i used in a registration, adapted from Finnegan et al. [5].

Algorithm 1 Constructing distance-preservation maps (DP_i).

for each ROI_i **do**
  $\text{M}_i \leftarrow$ Mask of ROI_i
  $\text{MD}_i \leftarrow$ Maurer distance map of M_i
  $\text{MD}_i \leftarrow$ Set values of MD_i not in M_i to 0
  $\text{MD}_i \leftarrow$ Normalize[a] MD_i
  $\text{DP}_i \leftarrow$ Set values of MD_i not in M_i to -1
end for

[a] Min-max normalization: $(\text{X}-\text{X}_\text{min})/(\text{X}_\text{max}-\text{X}_\text{min})$

Fixed masks were constructed to focus the registration on aligning prostatic regions. For image data, the fixed mask was the bounding box of ROI_F dilated by 6 mm in the x-y plane and 10 mm in the z direction. For DP-maps,

i.e. aligning DP_M with DP_F, the fixed mask was the bounding box of ROI_F dilated by 8 mm in the x-y plane and 10 mm in the z direction.

The registration algorithm uses a three-level multiresolution approach with Gaussian image pyramids for the moving and fixed images; $\sigma = 2$, 1 and 0.5 voxels in each direction x, y, z for the three resolution levels respectively. We used *random coordinate* sampling, thus bypassing the time savings of a Gaussian pyramid with downsampling [2], and adaptive stochastic gradient descent optimization for $n_{\text{iter}} = 2000$ the number of iterations.

For each iteration, the sampler selects a new voxel and draws 3000 samples from a $25\,\text{mm}^3$ square region around that voxel, hence the 'local' in LMI.

We used a linear interpolator during optimization, and nearest neighbour interpolation in the resampling. We employed a *recursive B-spline transformation* [2], defined by a multigrid of control points with a spacing of 80, 40 and 20 mm for the first, second and third resolution level, respectively.

3-D contrast limited adaptive histogram equalization (MCLAHE) [6] was used to bring the moving and fixed image data into similar dynamic range within the prostate regions before registration. This also has the effect of assigning a larger part of the available output bins for gray levels that many voxels in the input had. For the MCLAHE we used a default kernel size, set a clip limit of 0.02, and assigned 64 gray-level bins to the local histograms. The same number of bins were also used for the registration algorithm at all resolution levels.

Image acquisition and processing

This section provides additional details surrounding image acquisitions and how registrations were applied.

Histopathology and ex-vivo imaging

Figure S1 illustrates the pipeline for obtaining and registering the histopathology to the in-vivo T2w image. The registration process follows our previously described methodology [7], except that we have updated the non-rigid registration such that it now also consider the preservation of internal distances [5].

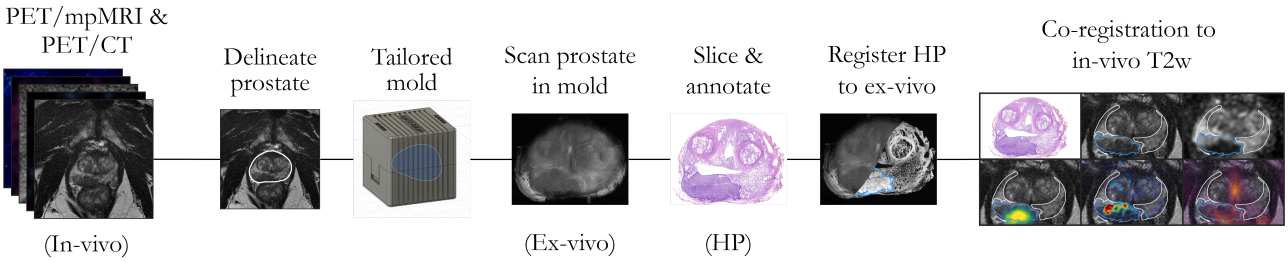

Fig. S1 Registration of histopathology (HP) to in-vivo, using ex-vivo imaging as an intermediate step.

DWI

Axial echo-planar DWI was acquired using three b-values: $b_0 = 0\,\text{s}\,\text{mm}^{-2}$, $b_{200} = 200\,\text{s}\,\text{mm}^{-2}$ and $b_{1000} = 1000\,\text{s}\,\text{mm}^{-2}$. The DWI was registered to the T2w assuming that all DWIs were aligned with $\text{DWI}(b_0)$. The registration was initialized by obtaining the translation $\mathcal{T}_T$ betwen ROI_{b_0} and ROI_{T2w}, and applying the resulting origin-to-origin translation vector $\Delta\mathbf{x}$ to all DWIs. Next we found the $\mathcal{T}_N$ between the T2w and the translated $\text{DWI}(b_0)$, and applied that to all DWIs. ADC maps were obtained pixelwise by solving the linear system of equations of the monoexpenontial decay model for two measurements,

$$\begin{aligned} \text{DWI}(b_{200}) &= \text{DWI}(b_0)e^{-\text{ADC}b_{200}} \\ \text{DWI}(b_{1000}) &= \text{DWI}(b_0)e^{-\text{ADC}b_{1000}}. \end{aligned} \tag{1}$$

Eliminating $S(b_0)$ and solving for ADC yields

$$\text{ADC} = \frac{\ln \text{DWI}(b_{200}) - \ln \text{DWI}(b_{1000})}{1000 - 200}. \tag{2}$$

DCE

Axial DCE images were obtained by a Fast Spoiled Gradient Recalled Echo (FSPGR) T1-weighted (T1w) sequence as 50 frames over 8 minutes, using a gadolinium (GD)-based contrast agent (CA) ($0.2\,\text{ml}\,\text{kg}^{-1}$ or maximum 20 ml Dotarem, $279.3\,\text{mg}\,\text{ml}^{-1}$, Guerbet, Villepinte, France), injected intravenously. The frames were motion-corrected by using the rigid registration method $\mathcal{T}_R$, with 16, 32 and 64 number of gray-level bins for the three resolution

levels, respectively. CA maps were calculated using T1-maps, the FSPGR before the onset of CA as baseline, and the complete motion-corrected FSPGR+GD as a dynamic series [8, 9]. T1 maps were estimated by the variable flip angle method [10], using 2° and 15° flip angles. Repetition times were in the range 4.03–4.49 ms and echo times in the range 1.82–1.90 ms. The T1-maps where rigidly registered against the motion-corrected FSPGR+GD using the rigid registration $\mathcal{T}_R$ with 32 gray-level bins for all resolution levels. Arterial input functions were determined for each patient from the CA maps by drawing ROIs in the deep and/or superficial femoral artery in the frame showing maximal enhancement and in a slice close to the centre of the prostate, but distal to inflowing spins to approach a steady state [11]. Using the AIF to quantify image intensities, the volume transfer constant (K^{trans}) could be obtained by fitting the CA concentration to a three-parameter Kety model [12]. K^{trans} was registered to the T2w image by the same procedure as explained for the DWI(b_0).

PET data

The CT from the Acetate-PET/CT was registered to the T2w, and the resulting transforms were applied to the Acetate-PET data. We used the rigid registration method $\mathcal{T}_R$ with 32 gray-level bins for all resolution levels and resampled the result to the T2w image using nearest neighbour interpolation. PSMA-PET was assumed to be aligned with the T2w image, and merely resampled by nearest-neighbour interpolation.

Classification algorithm

Receiver operating characteristics (ROCs) were obtained using a fast implementation of DeLong's algorithm [13]. This algorithm takes as inputs *probability estimates* and a ground truth (*target*). When using multiple predictors, the probability estimates were obtained by fitting logistic regression models using the Scikit-learn library of Python [14] with default parameters and standard scaling. For instance,

```
from sklearn.datasets import load_breast_cancer
from sklearn.linear_model import LogisticRegression
from sklearn.preprocessing import StandardScaler

dataset = load_breast_cancer(return_X_y=True)
# Example with two predictors
X2, target = dataset[0][:, 8:10], dataset[1]
# Standard scaling
sc = StandardScaler()
X = sc.fit_transform(X2)
# Model
clf = LogisticRegression().fit(X, target)
probability_estimates = clf.predict_proba(X)[::, 1]
```

When only one predictor is used, cut-off values associated with the maximum Youden thresholds are obtained by a naïve implementation. The ROC results are numerically equivalent to the results from logistic regression.

```
import numpy as np
X1, target = dataset[0][:, 9:10].flatten(), dataset[1]
# If larger X's are expected to be associated with target == 1.
predictions = np.array([X1 > X_i for X_i in X1])
cms = np.array([np.bincount(target * 2 + prediction, minlength=4)
                for prediction in predictions])
sens = cms[:, 3] / (cms[:, 3] + cms[:, 2])
spec = cms[:, 0] / (cms[:, 0] + cms[:, 1])
max_Youden_idx = np.argmax(sens + spec)
Youden_threshold = X1[max_Youden_idx]
```

Adjustments for size were done using the *statsmodels* package [15], similarly to

```
import statsmodels.api as sm
model_1 = sm.Logit(target, X1)
model_2 = sm.Logit(target, X2)
result_1 = model_1.fit()
result_2 = model_2.fit()
print(result_1.summary())
print(result_2.summary())
```

Supplementary Figures

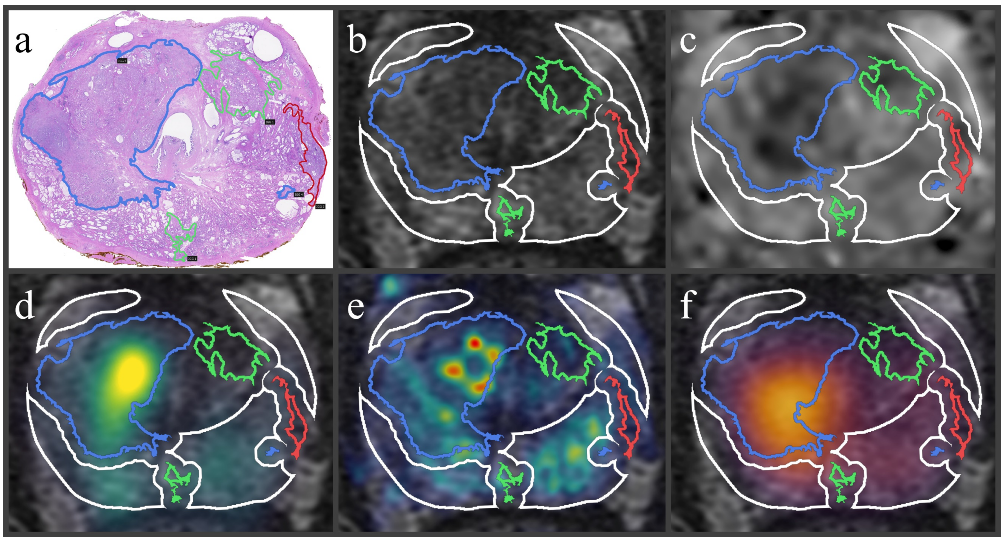

Supplementary Fig. 1 – (a) Histological section showing the contour of two IGG 4 lesions (blue), one IGG 3 lesion (red) and two IGG 1 lesion (green). (b) T2w with the registered lesions and the non-malignant PZ (white), where voxels in the PZ closer than 1 mm from lesions had been removed. The T2w image served as a common frame of reference for the histopathology, ADC (c), PSMA-PET (d), K^{trans} (e) and Acetate-PET (f). IGG = International Society of Urological Pathology grade group; T2w = T2-weighted; PZ = peripheral zone; ADC = apparent diffusion coefficient; PSMA = prostate-specific membrane antigen; PET = positron emission tomography; K^{trans} = volume transfer constant.

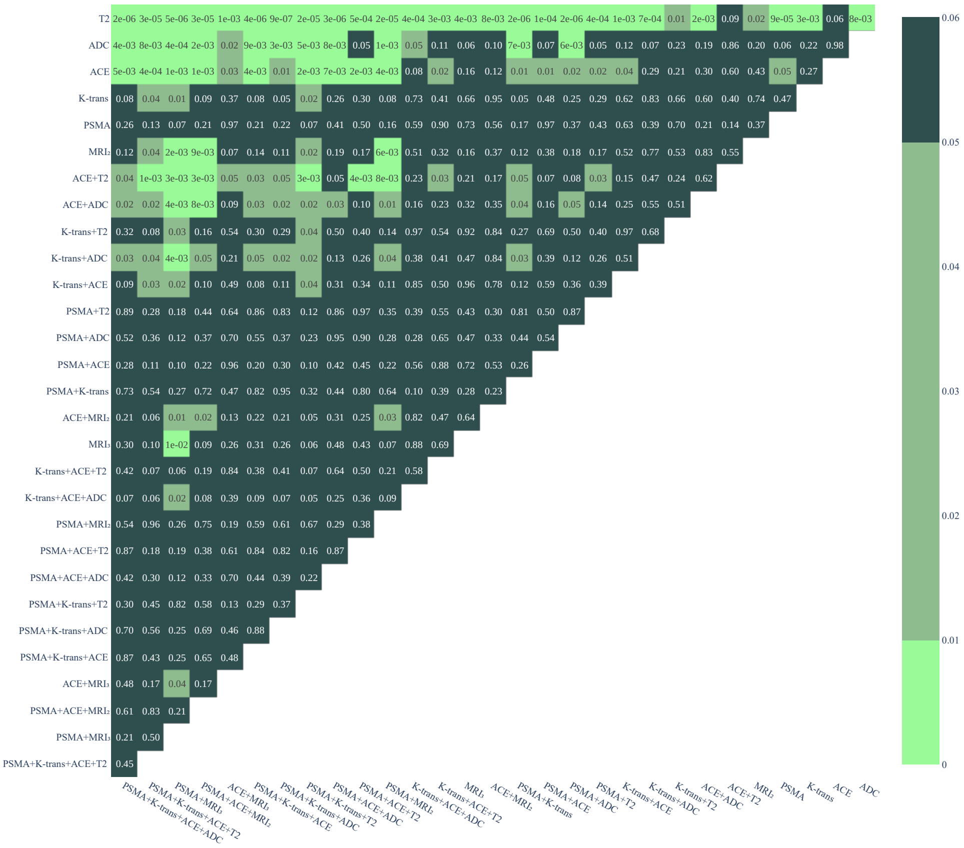

Supplementary Fig. 2 – P-value matrix for comparisons of models discriminating between International Society of Urological Pathology grade groups (IGG) 3 and IGG 2. Statistical significance is indicated by the two brighter shades ($p < 0.05$ and $p < 0.01$, in order of increasing brightness).
PSMA = maximum standardized uptake value per lesion for [^{68}Ga]PSMA-11; ACE = maximum standardized uptake value per lesion for [^{11}C]Acetate; K-trans = maximum volume transfer constant per lesion, divided by the mean volume transfer constant in the non-malignant peripheral zone (PZ) of the patient; ADC = median apparent diffusion coefficient per lesion, divided by the mean apparent diffusion coefficient in the non-malignant PZ of the patient; T2 = median image intensity per lesion in the T2w image, divided by the mean image intensity in the non-malignant PZ of the patient; MRI_3 = Combining K-trans, ADC, and T2 as individual variables in the logistic regression model; MRI_2 = Combining only the latter two; The plus sign (+) is also used to indicate that multiple variables have been combined; n = 194 independent lesions for IGG ≥ 3 vs. IGG ≤ 2 and n = 123 for IGG 3 vs. IGG 2.

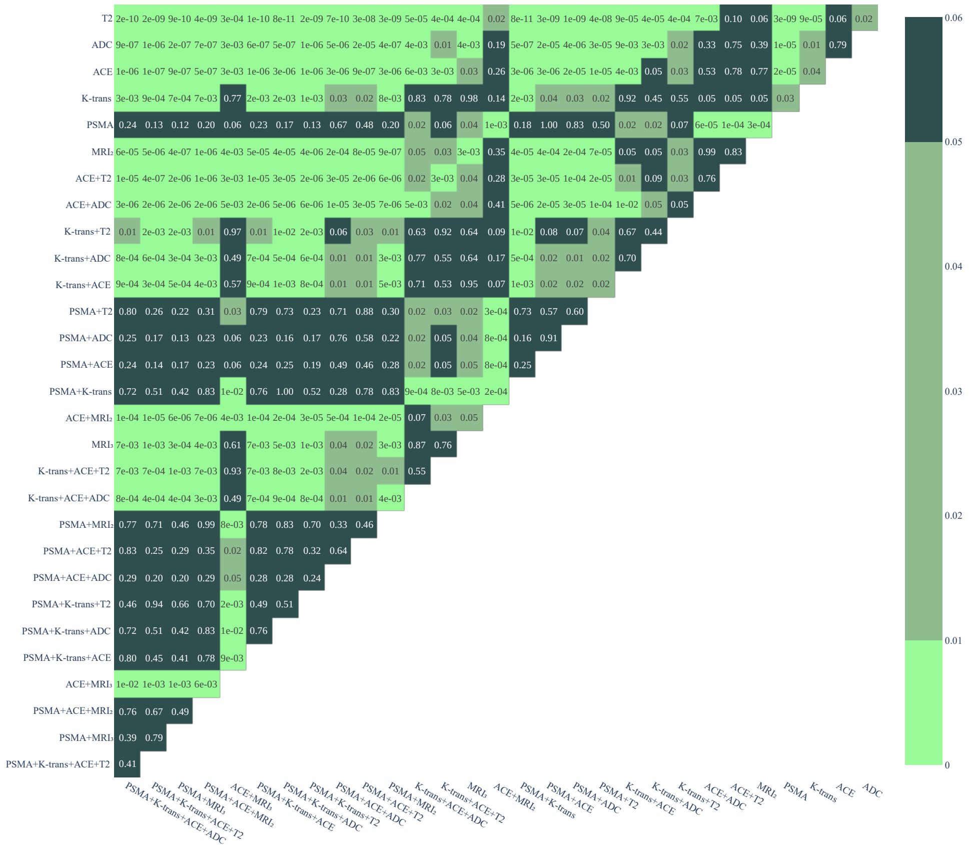

Supplementary Fig. 3 – P-value matrix for comparison of models discriminating between International Society of Urological Pathology grade groups (IGG) ≥ 3 and IGG ≤ 2. Statistical significance is indicated by the two brighter shades ($p < 0.05$ and $p < 0.01$, in order of increasing brightness).
PSMA = maximum standardized uptake value per lesion for [^{68}Ga]PSMA-11; ACE = maximum standardized uptake value per lesion for [^{11}C]Acetate; K-trans = maximum volume transfer constant per lesion, divided by the mean volume transfer constant in the non-malignant peripheral zone (PZ) of the patient; ADC = median apparent diffusion coefficient per lesion, divided by the mean apparent diffusion coefficient in the non-malignant PZ of the patient; T2 = median image intensity per lesion in the T2w image, divided by the mean image intensity in the non-malignant PZ of the patient; MRI_3 = Combining K-trans, ADC, and T2 as individual variables in the logistic regression model; MRI_2 = Combining only the latter two; The plus sign (+) is also used to indicate that multiple variables have been combined; n = 194 independent lesions for IGG ≥ 3 vs. IGG ≤ 2 and n = 123 for IGG 3 vs. IGG 2.

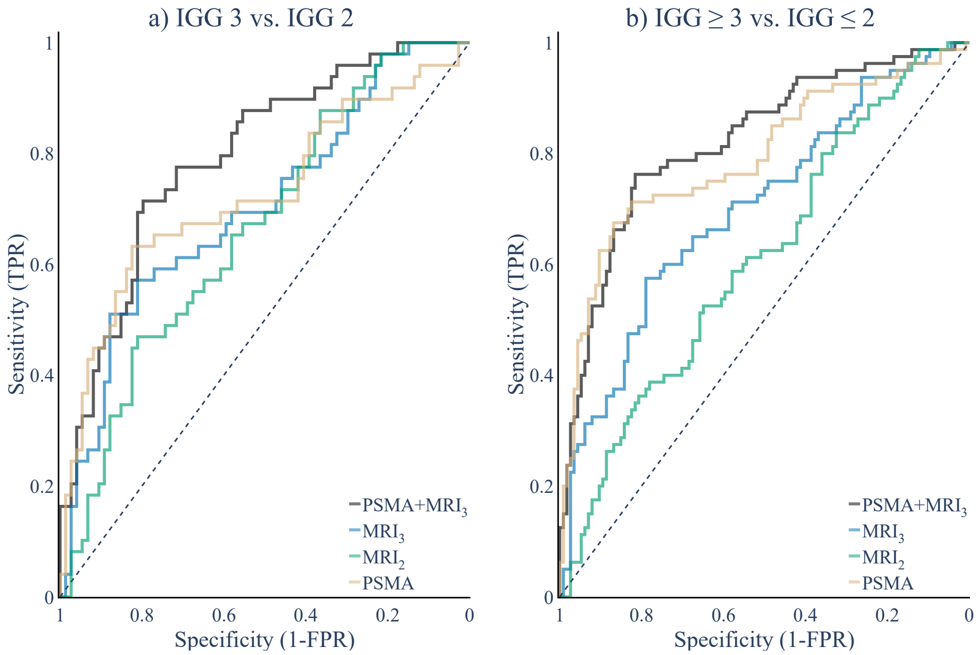

Supplementary Fig. 4 – A selection of receiver operating characteristic (ROC) curves, using one or several modalities in logistic regression models to discriminate between International Society of Urological Pathology grade groups (IGG), where a correctly classified higher-grade lesion was considered a true positive. PSMA = maximum standardized uptake value per lesion for [^{68}Ga]PSMA-11; K-trans = maximum volume transfer constant per lesion, divided by the mean volume transfer constant in the non-malignant peripheral zone (PZ) of the patient; ADC = median apparent diffusion coefficient per lesion, divided by the mean apparent diffusion coefficient in the non-malignant PZ of the patient; T2 = median image intensity per lesion in the T2w image, divided by the mean image intensity in the non-malignant PZ of the patient; MRI_2 = fitting a logistic regression model combining ADC and T2 as individual variables; MRI_3 = combining K-trans, ADC, and T2; PSMA+MRI_3 = combining PSMA and the components of MRI_3 ;n = 194 independent lesions for IGG ≥ 3 vs. IGG ≤ 2 and n = 123 for IGG 3 vs. IGG 2.

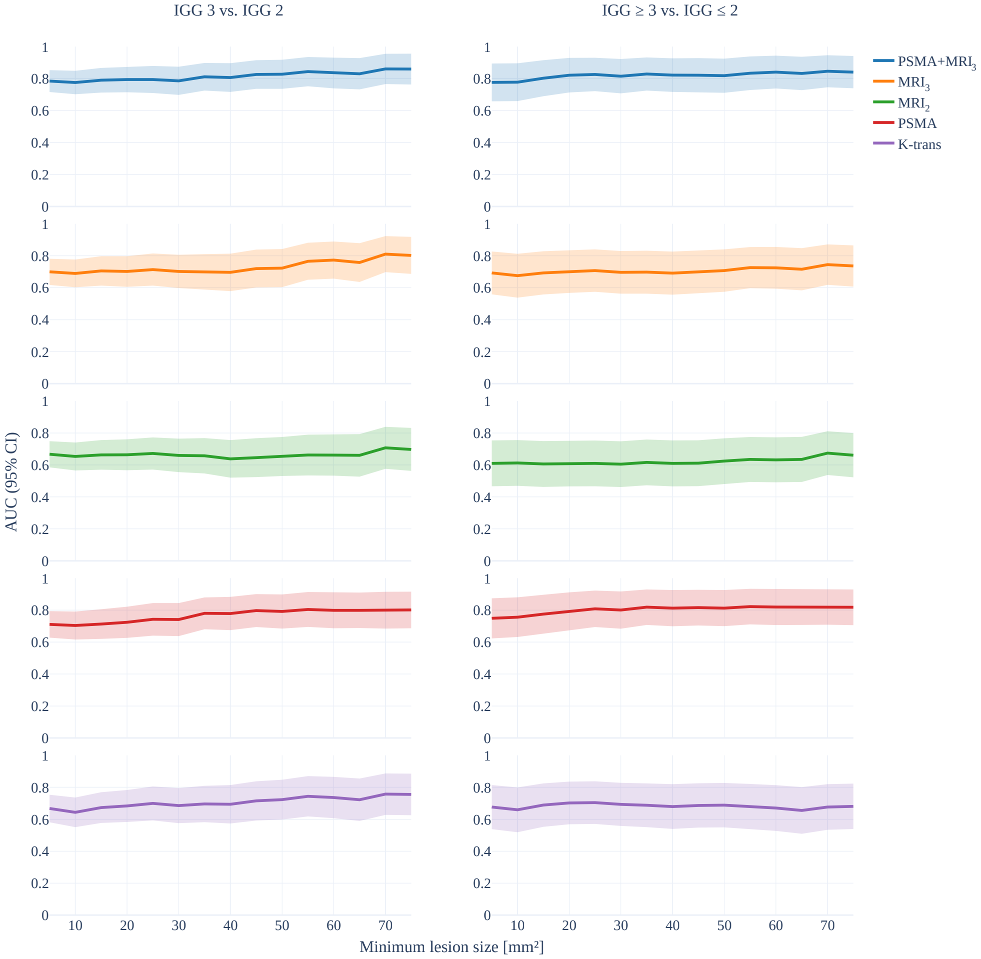

Supplementary Fig. 5 – Area under the receiver operating characteristics curves (AUC) with 95% confidence intervals (CI) using one or several modalities to discriminate between International Society of Urological Pathology grade groups (IGG), where a correctly classified higher-grade lesion was considered a true positive. The x-axis refers to the lower bound of lesion sizes included when constructing a particular AUC.
PSMA = maximum standardized uptake value per lesion for [^{68}Ga]PSMA-11; K-trans = maximum volume transfer constant per lesion, divided by the mean volume transfer constant in the non-malignant peripheral zone (PZ) of the patient; ADC = median apparent diffusion coefficient per lesion, divided by the mean apparent diffusion coefficient in the non-malignant PZ of the patient; T2 = median image intensity per lesion in the T2w image, divided by the mean image intensity in the non-malignant PZ of the patient; MRI_2 = fitting a logistic regression model combining ADC and T2 as individual variables; MRI_3 = combining K-trans, ADC, and T2; PSMA+MRI_3 = combining PSMA and the components of MRI_3; n = 194 independent lesions for IGG ≥ 3 vs. IGG ≤ 2 and n = 123 for IGG 3 vs. IGG 2.

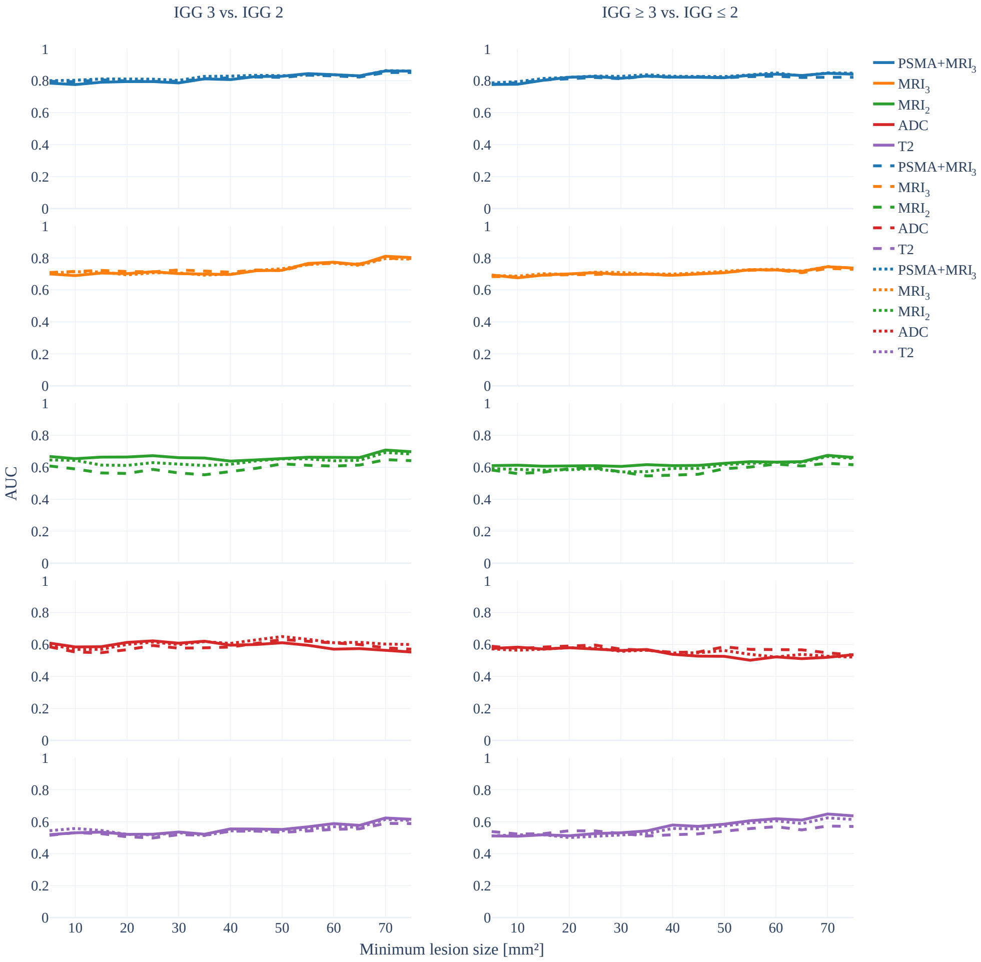

Supplementary Fig. 6 – Area under the receiver operating characteristics curves (AUC) using one or several modalities to discriminate between International Society of Urological Pathology grade groups (IGG), where a correctly classified higher-grade lesion was considered a true positive. The x-axis refers to the lower bound of lesion sizes included when constructing a particular AUC. Solid lines indicate that median values for ADC and T2 have been used, as opposed to minimum (dashed lines) or near-minimum (5th percentile) (dotted lines). PSMA = maximum standardized uptake value per lesion for [^{68}Ga]PSMA-11; K-trans = maximum volume transfer constant per lesion, divided by the mean volume transfer constant in the non-malignant peripheral zone (PZ) of the patient; ADC = median apparent diffusion coefficient per lesion, divided by the mean apparent diffusion coefficient in the non-malignant PZ of the patient; T2 = median image intensity per lesion in the T2w image, divided by the mean image intensity in the non-malignant PZ of the patient; MRI_2 = fitting a logistic regression model combining ADC and T2 as individual variables; MRI_3 = combining K-trans, ADC, and T2; PSMA+MRI_3 = combining PSMA and the components of MRI_3; n = 194 independent lesions for IGG ≥ 3 vs. IGG ≤ 2 and n = 123 for IGG 3 vs. IGG 2.

Supplementary Tables

Supplementary Table 1 – Area under the receiver operating characteristic curve (AUC) with 95% confidence intervals (CI). A correctly classified higher-grade lesion is considered a true positive. Grades are defined in terms of International Society of Urological Pathology grade groups (IGG).

	**AUC [95% CI]**	
**Modalities**	**IGG 3 vs. IGG 2**	**IGG ≥ 3 vs. IGG ≤ 2**
PSMA+$\widetilde{K}^{trans}$+ACE+$\widetilde{ADC}$	0.76 [0.66–0.85]	0.81 [0.74–0.87]
PSMA+$\widetilde{K}^{trans}$+ACE+$\widetilde{T2}$	0.78 [0.70–0.86]	0.82 [0.76–0.88]
PSMA+MRI$_3$	0.79 [0.71–0.87]	0.82 [0.76–0.88]
PSMA+ACE+MRI$_2$	0.77 [0.69–0.86]	0.81 [0.75–0.88]
Ktrans+ACE+MRI$_2$	0.72 [0.63–0.81]	0.71 [0.63–0.79]
PSMA+$\widetilde{K}^{trans}$+ACE	0.76 [0.66–0.85]	0.81 [0.74–0.88]
PSMA+$\widetilde{K}^{trans}$+$\widetilde{ADC}$	0.76 [0.66–0.85]	0.81 [0.74–0.88]
PSMA+$\widetilde{K}^{trans}$+$\widetilde{T2}$	0.79 [0.71–0.87]	0.82 [0.76–0.88]
PSMA+ACE+$\widetilde{ADC}$	0.74 [0.64–0.84]	0.80 [0.73–0.86]
PSMA+ACE+$\widetilde{T2}$	0.75 [0.66–0.84]	0.80 [0.74–0.87]
PSMA+MRI$_2$	0.78 [0.70–0.86]	0.81 [0.75–0.88]
$\widetilde{K}^{trans}$+ACE+$\widetilde{ADC}$	0.70 [0.60–0.80]	0.70 [0.62–0.78]
$\widetilde{K}^{trans}$+ACE+$\widetilde{T2}$	0.72 [0.62–0.81]	0.71 [0.63–0.79]
MRI$_3$	0.70 [0.61–0.80]	0.70 [0.63–0.78]
ACE+MRI$_2$	0.69 [0.59–0.78]	0.63 [0.55–0.71]
PSMA+$\widetilde{K}^{trans}$	0.76 [0.66–0.85]	0.81 [0.74–0.88]
PSMA+ACE	0.73 [0.63–0.82]	0.79 [0.72–0.86]
PSMA+$\widetilde{ADC}$	0.74 [0.65–0.84]	0.79 [0.72–0.86]
PSMA+$\widetilde{T2}$	0.75 [0.66–0.84]	0.80 [0.74–0.87]
$\widetilde{K}^{trans}$+ACE	0.70 [0.60–0.80]	0.70 [0.62–0.78]
$\widetilde{K}^{trans}$+$\widetilde{ADC}$	0.68 [0.58–0.78]	0.69 [0.61–0.77]
$\widetilde{K}^{trans}$+$\widetilde{T2}$	0.70 [0.60–0.80]	0.71 [0.64–0.79]
ACE+$\widetilde{ADC}$	0.65 [0.55–0.75]	0.61 [0.53–0.69]
ACE+$\widetilde{T2}$	0.63 [0.52–0.73]	0.60 [0.52–0.68]
MRI$_2$	0.66 [0.57–0.76]	0.61 [0.53–0.69]
PSMA	0.72 [0.63–0.82]	0.79 [0.72–0.86]
$\widetilde{K}^{trans}$	0.68 [0.58–0.78]	0.70 [0.63–0.78]
ACE	0.61 [0.51–0.71]	0.59 [0.51–0.67]
$\widetilde{ADC}$	0.61 [0.51–0.71]	0.58 [0.49–0.66]
$\widetilde{T2}$	0.48 [0.38–0.58]	0.49 [0.41–0.57]

PSMA = maximum standardized uptake value per lesion for [^{68}Ga]PSMA-11; ACE = maximum standardized uptake value per lesion for [^{11}C]Acetate; $\widetilde{K}^{trans}$ = maximum volume transfer constant per lesion, divided by the mean volume transfer constant in the non-malignant peripheral zone (PZ) of the patient; $\widetilde{ADC}$ = median apparent diffusion coefficient per lesion, divided by the mean apparent diffusion coefficient in the non-malignant PZ of the patient; $\widetilde{T2}$ = median image intensity per lesion in the T2w image, divided by the mean image intensity in the non-malignant PZ of the patient. MRI$_3$ = fitting a logistic regression model combining $\widetilde{K}^{trans}$, $\widetilde{ADC}$, and $\widetilde{T2}$; MRI$_2$ = combining $\widetilde{ADC}$, and $\widetilde{T2}$; The plus sign (+) also indicates that multiple variables have been combined; n = 194 independent lesions for IGG ≥ 3 vs. IGG ≤ 2 and n = 123 for IGG 3 vs. IGG 2.

Supplementary Table 2 – Area under the receiver operating characteristic curve (AUC) with 95% confidence intervals (CI). A correctly classified higher-grade lesion is considered a true positive. Grades are defined in terms of International Society of Urological Pathology grade groups (IGG). Illustrates the impact on results when varying the summary measures for apparent diffusion coefficient maps and T2-weighted images.

	**AUC [95% CI]**	
**Modalities**	**IGG 3 vs. IGG 2**	**IGG ≥ 3 vs. IGG ≤ 2**
PSMA + MRI_3		
Median	0.79 [0.71–0.87]	0.82 [0.76–0.88]
Minimum	0.80 [0.72–0.88]	0.81 [0.75–0.88]
Near-minimum (5th percentile)	0.82 [0.73–0.89]	0.82 [0.76–0.88]
MRI_3		
Median	0.70 [0.61–0.80]	0.70 [0.63–0.78]
Minimum	0.71 [0.62–0.81]	0.70 [0.62–0.77]
Near-minimum (5th percentile)	0.69 [0.60–0.79]	0.70 [0.62–0.77]
MRI_2		
Median	0.66 [0.57–0.76]	0.61[0.53–0.69]
Minimum	0.56 [0.46–0.66]	0.59[0.51–0.67]
Near-minimum (5th percentile)	0.61 [0.51–0.71]	0.58[0.50–0.67]
$\widetilde{ADC}$		
Median	0.61 [0.51–0.71]	0.58 [0.50–0.66]
Minimum	0.57 [0.46–0.67]	0.59 [0.51–0.67]
Near-minimum (5th percentile)	0.60 [0.50–0.70]	0.58 [0.50–0.66]
$\widetilde{T2}$		
Median	0.48 [0.38–0.58]	0.49 [0.41–0.57]
Minimum	0.49 [0.39–0.60]	0.46 [0.37–0.54]
Near-minimum (5th percentile)	0.48 [0.38–0.58]	0.50 [0.42–0.58]

Area under the receiver operating characteristics curves (AUC) with 95% confidence intervals (CI) using one or several modalities to discriminate between International Society of Urological Pathology grade groups (IGG), where a correctly classified higher-grade lesion was considered a true positive. PSMA = maximum standardized uptake value per lesion for [^{68}Ga]PSMA-11; $\widetilde{K}^{trans}$ = maximum volume transfer constant per lesion, divided by the mean volume transfer constant in the non-malignant peripheral zone (PZ) of the patient; $\widetilde{ADC}$ = median, minimum or near-minimum (5th percentile) apparent diffusion coefficient per lesion, divided by the mean apparent diffusion coefficient in the non-malignant PZ of the patient; $\widetilde{T2}$ = median, minimum or near-minimum (5th percentile) image intensity per lesion in the T2w image, divided by the mean image intensity in the non-malignant PZ of the patient. MRI_2 = fitting a logistic regression model combining $\widetilde{ADC}$ and $\widetilde{T2}$ as individual variables in the model; MRI_3 = combining $\widetilde{K}^{trans}$, $\widetilde{ADC}$, and $\widetilde{T2}$; PSMA+MRI_3 = combining PSMA and the components of MRI_3 as individual variables in the model; n = 194 independent lesions for IGG ≥ 3 vs. IGG ≤ 2 and n = 123 for IGG 3 vs. IGG 2.

Supplementary References

[1] NONPI Medical AB. MICE Toolkit — A software for medical image analysis. https://www.micetoolkit.com/ 2019. [Online; accessed 22-December-2022].

[2] Klein S, Staring M, Murphy K, Viergever MA, Pluim JP. Elastix: a toolbox for intensity-based medical image registration. *IEEE Trans Med Imaging* 2010;29:196 - 205.

[3] Klein S, Pluim JPW, Staring M, Viergever MA. Adaptive Stochastic Gradient Descent Optimisation for Image Registration. *Int J Comput Vis* 2008;81:227.

[4] Klein S, van der Heide UA, Lips IM, van Vulpen M, Staring M, Pluim JPW. Automatic segmentation of the prostate in 3D MR images by atlas matching using localized mutual information. *Med Phys* 2008;35:1407-1417.

[5] Finnegan RN, Reynolds HM, Ebert MA, et al. A statistical, voxelised model of prostate cancer for biologically optimised radiotherapy. *Phys Imaging Radiat Oncol* 2022;21:136-145.

[6] Stimper V, Bauer S, Ernstorfer R, Schölkopf B, Xian RP. Multidimensional Contrast Limited Adaptive Histogram Equalization. *IEEE Access* 2019;7:165437-165447.

[7] Sandgren K, Nilsson E, Keeratijarut Lindberg A, et al. Registration of histopathology to magnetic resonance imaging of prostate cancer. *Phys Imaging Radiat Oncol* 2021;18:19-25.

[8] Blüml S, Schad LR, Stepanow B, Lorenz WJ. Spin-lattice relaxation time measurement by means of a TurboFLASH technique. *Magn Reson Med* 1993;30:289-295.

[9] Schabel MC, Parker DL. Uncertainty and bias in contrast concentration measurements using spoiled gradient echo pulse sequences. *Phys Med Biol* 2008;53:2345–2373.

[10] Deoni SC, Peters TM, Rutt BK. High-resolution T1 and T2 mapping of the brain in a clinically acceptable time with DESPOT1 and DESPOT2. *Magn Reson Med* 2005;53:237–241.

[11] Roberts C, Little R, Watson Y, Zhao S, Buckley DL, Parker GJM. The effect of blood inflow and B1-field inhomogeneity on measurement of the arterial input function in axial 3D spoiled gradient echo dynamic contrast-enhanced MRI. *Magn Reson Med* 2011;65:108-119.

[12] Murase K. Efficient method for calculating kinetic parameters using T1-weighted dynamic contrast-enhanced magnetic resonance imaging. *Magn Reson Med* 2004;51:858-862.

[13] Sun X, Xu W. Fast implementation of DeLong's algorithm for comparing the areas under correlated receiver operating characteristic curves. *IEEE Signal Process Lett* 2014;21:1389–1393.

[14] Pedregosa F, Varoquaux G, Gramfort A, et al. Scikit-learn: Machine Learning in Python. *Journal of Machine Learning Research* 2011;12:2825–2830.

[15] Seabold S, Perktold J. Statsmodels: Econometric and statistical modeling with python. in *9th Python in Science Conference* 2010.
