# Supplementary material for: The grade of individual prostate cancer lesions predicted by magnetic resonance imaging and positron emission tomography
Source: Commun Med (Lond). 2023 Nov 9;3:164. doi: 10.1038/s43856-023-00394-7 (PMC10636013; doi:10.1038/s43856-023-00394-7)
Supplement: Supplementary file 2 — Reporting Summary [file 43856_2023_394_MOESM2_ESM.pdf]

Corresponding author(s): Erik Nilsson
Last updated by author(s): Oct 7, 2023

# Reporting Summary

Nature Portfolio wishes to improve the reproducibility of the work that we publish. This form provides structure for consistency and transparency in reporting. For further information on Nature Portfolio policies, see our Editorial Policies and the Editorial Policy Checklist.

## Statistics

For all statistical analyses, confirm that the following items are present in the figure legend, table legend, main text, or Methods section.

| n/a | Confirmed | |
|---|---|---|
| ☐ | ☒ | The exact sample size (*n*) for each experimental group/condition, given as a discrete number and unit of measurement |
| ☐ | ☒ | A statement on whether measurements were taken from distinct samples or whether the same sample was measured repeatedly |
| ☐ | ☒ | The statistical test(s) used AND whether they are one- or two-sided<br>*Only common tests should be described solely by name; describe more complex techniques in the Methods section.* |
| ☒ | ☐ | A description of all covariates tested |
| ☐ | ☒ | A description of any assumptions or corrections, such as tests of normality and adjustment for multiple comparisons |
| ☐ | ☒ | A full description of the statistical parameters including central tendency (e.g. means) or other basic estimates (e.g. regression coefficient) AND variation (e.g. standard deviation) or associated estimates of uncertainty (e.g. confidence intervals) |
| ☐ | ☒ | For null hypothesis testing, the test statistic (e.g. *F*, *t*, *r*) with confidence intervals, effect sizes, degrees of freedom and *P* value noted<br>*Give P values as exact values whenever suitable.* |
| ☒ | ☐ | For Bayesian analysis, information on the choice of priors and Markov chain Monte Carlo settings |
| ☒ | ☐ | For hierarchical and complex designs, identification of the appropriate level for tests and full reporting of outcomes |
| ☒ | ☐ | Estimates of effect sizes (e.g. Cohen's *d*, Pearson's *r*), indicating how they were calculated |

*Our web collection on statistics for biologists contains articles on many of the points above.*

## Software and code

Policy information about availability of computer code

| | |
|---|---|
| Data collection | No software was used |
| Data analysis | RayStation v.8.99.30.16 (RaySearch Laboratories, Stockholm, Sweden)<br>MICE Toolkit v.2021.2.1 (Medical Interactive Creative Environment, NONPI Medical, Umeå, Sweden)<br>Scikit-learn v.1.0.2: Machine Learning in Python v.3.9.5<br>Statsmodels v.0.14.0: Econometric and statistical modeling with python |

For manuscripts utilizing custom algorithms or software that are central to the research but not yet described in published literature, software must be made available to editors and reviewers. We strongly encourage code deposition in a community repository (e.g. GitHub). See the Nature Portfolio guidelines for submitting code & software for further information.

## Data

Policy information about availability of data

All manuscripts must include a data availability statement. This statement should provide the following information, where applicable:

- Accession codes, unique identifiers, or web links for publicly available datasets
- A description of any restrictions on data availability
- For clinical datasets or third party data, please ensure that the statement adheres to our policy

The data that support the findings of this study are available from the corresponding author upon reasonable request.

## Human research participants

Policy information about studies involving human research participants and Sex and Gender in Research.

| | |
|---|---|
| Reporting on sex and gender | The results of this study applies to patients with confirmed prostate cancer. As such, they were assigned as males (sex). We did not collect sex- and gender-based information nor did we perform any sex- and gender-based analysis. Effects caused by social and cultural circumstances was beyond the scope of this study. Androgen deprivation therapy and/or castration would have excluded a patient from the study. |
| Population characteristics | Median age 63 years (range 45–76 years). All patients presented an elevated prostatic-specific antigen (PSA) (median PSA: 6.3 ng/ml; range: 2.9–13.3 ng/ml), biopsy-verified intermediate and high-risk PCa (IGG ≥ 2, at least 2 mo prior to surgery), and were planned for laparoscopic radical prostatectomy. |
| Recruitment | Patients were included consecutively. No potential bias related to self-selection has been identified. |
| Ethics oversight | Regional Ethics Board and the Radiation Protection Committee at the University Hospital of Northern Sweden |

Note that full information on the approval of the study protocol must also be provided in the manuscript.

# Field-specific reporting

Please select the one below that is the best fit for your research. If you are not sure, read the appropriate sections before making your selection.

☒ Life sciences ☐ Behavioural & social sciences ☐ Ecological, evolutionary & environmental sciences

For a reference copy of the document with all sections, see nature.com/documents/nr-reporting-summary-flat.pdf

# Life sciences study design

All studies must disclose on these points even when the disclosure is negative.

| | |
|---|---|
| Sample size | 55 patients yielding 600 lesions. The results are based on the 194 lesions larger than 20 mm² No sample-size calculation was performed. The threshold in size is related to the registration uncertainty. |
| Data exclusions | No meaningful image characteristics can be determined for lesions of sizes comparable to the registration uncertainty. The risk of adding noise to the analysis was weighed against the risk of introducing bias towards larger lesions, and we decided on a threshold of in-plane area ≥20 mm². This corresponds to the area of a circle with a radius r ≈ 2.5 mm, which is roughly twice the estimated registration uncertainty. |
| Replication | We intentionally chose to evaluate rudimentary image characteristics in order to improve reproducibility. |
| Randomization | 55 consecutive patients were included in the study. They were anonymized and given a randomized identifier. |
| Blinding | Not relevant. The registration process of the histopathology to the in-vivo image data was conducted before the analysis of image characteristics within the registered lesions. |

# Reporting for specific materials, systems and methods

We require information from authors about some types of materials, experimental systems and methods used in many studies. Here, indicate whether each material, system or method listed is relevant to your study. If you are not sure if a list item applies to your research, read the appropriate section before selecting a response.

### Materials & experimental systems

| n/a | Involved in the study | |
|---|---|---|
| ☒ | ☐ | Antibodies |
| ☒ | ☐ | Eukaryotic cell lines |
| ☒ | ☐ | Palaeontology and archaeology |
| ☒ | ☐ | Animals and other organisms |
| ☐ | ☒ | Clinical data |
| ☒ | ☐ | Dual use research of concern |

### Methods

| n/a | Involved in the study | |
|---|---|---|
| ☒ | ☐ | ChIP-seq |
| ☒ | ☐ | Flow cytometry |
| ☒ | ☐ | MRI-based neuroimaging |

## Clinical data

Policy information about clinical studies

All manuscripts should comply with the ICMJE guidelines for publication of clinical research and a completed CONSORT checklist must be included with all submissions.

| | |
|---|---|
| Clinical trial registration | EudraCT Number: 2015-005046-55 |
| Study protocol | https://euclinicaltrials.eu/ |
| Data collection | The data was collected at the University Hospital of Northern Sweden, Department of Radiation Sciences and the Department of Urology. |
| Outcomes | The diagnostic potential of positron emission tomography and multiparametric MRI was investigated with measures of the International Society of Urological Pathology Grade Groups, and the measures were evaluated with histopathological reference standards. |
